# Inhomogeneity correction and the analytic anisotropic algorithm

**DOI:** 10.1120/jacmp.v9i2.2786

**Published:** 2008-05-01

**Authors:** Don Robinson

**Affiliations:** ^1^ Department of Medical Physics Cross Cancer Institution Edmonton Alberta Canada; ^2^ Department of Oncology University of Alberta Edmonton Alberta Canada

**Keywords:** Inhomogeneity correction factor, analytic anisotropic algorithm, AAA

## Abstract

The ability of the analytic anisotropic algorithm (AAA), a superposition– convolution algorithm implemented in the Eclipse (Varian Medical Systems, Palo Alto, CA) treatment planning system (TPS), to accurately account for the presence of inhomogeneities in simple geometries is examined. The goal of 2% accuracy, as set out by the American Association of Physicists in Medicine Task Group 65, serves as a useful benchmark against which to evaluate the inhomogeneity correction capabilities of this treatment planning algorithm. A planar geometry phantom consisting of upper and lower layers of Solid Water (Gammex rmi, Middleton, WI) separated by a heterogeneity region of variable thickness, is modeled within the Eclipse TPS. Results obtained with the AAA are compared with experimental measurements. Seven different materials, spanning the range from air to aluminum, constitute the inhomogeneity layer. In general, the AAA overpredicts dose beyond low‐density regions and underpredicts dose distal to volumes of high density. In many cases, the deviation between the AAA and experimental results exceeds the Task Group 65 target of 2%. The source of these deviations appears to arise from an inability of the AAA to correctly account for altered attenuation along primary ray paths.

PACS number: 87.53.Tf

## I. INTRODUCTION

Maximizing the therapeutic benefit of radiation treatments critically depends on delivering the prescribed dose to the entire planning target volume while minimizing the dose received by surrounding uninvolved tissues. Achieving this goal requires accurate spatial localization of all relevant structures and accurate calculation of the absorbed dose.

The heterogeneous composition of the human body presents numerous tissues types and cavities with widely differing radiologic properties. These include lungs, bones, teeth, sinuses, and nasal and oral cavities. Optimization of radiotherapeutic impact requires correct accounting for this heterogeneity so that absorbed dose may be accurately determined in all irradiated tissues. In their review of tissue inhomogeneity corrections, Task Group 65 (TG‐65) of the American Association of Physicists in Medicine (AAPM) recognized that properly accounting for tissue heterogeneity “is an essential component of dose optimization and the objective analysis of clinical results, especially with the advent of 3D precision conformal radiotherapy and the extension of intensity‐modulated radiation therapy (IMRT) treatments to structures that have not been irradiated before.”^(1)^ With regard to the goal of achieving optimum radiotherapy outcomes, TG‐65 concluded that “the general principle of 3% accuracy in dose delivery with the corresponding need for better than 2% accuracy in correcting for inhomogeneities is a reasonable, albeit challenging, goal.”^(1)^

The algorithms employed by radiotherapy treatment planning systems (TPSs) to model the distribution of dose in clinically relevant situations have evolved over the years from simple computations based on two‐dimensional look‐up tables to more sophisticated three‐dimensional (3D) approaches. This evolution has been aided by the development of faster and more powerful computers.

In 2005, the analytic anisotropic algorithm (AAA) for dose calculation was released within an established commercial TPS (Eclipse: Varian Medical Systems, Palo Alto, CA). The convolution–superposition approach used by the AAA to model the distribution of radiation deposited dose has proven reasonably successful at handling a wide range of clinically relevant situations, including heterogeneous geometries.

Accounting for the presence of tissue heterogeneity has traditionally assumed two general approaches. The more traditional form first calculates a relative dose distribution within a medium of homogeneous water‐equivalent composition. This initial distribution is then transformed through the application of inhomogeneity correction factors (ICFs). The relative distributions are converted to absolute dose by means of a suitable calibration factor defined at a reference point in water.

Various means of calculating ICFs—ranging from simple one‐dimensional approaches such as the percent per centimeter method^(^ ^2^ ^,^ ^3^ ^)^ to more sophisticated 3D techniques such as the equivalent tissue–air ratio (ETAR) method^(4)^—have been extensively discussed in the literature^(^ ^5^ ^–^ ^9^ ^)^ and are summarized in report 24 from the International Commission on Radiation Units and Measurements.^(10)^ Varying degrees of accuracy have been achieved with these methods, depending on the geometry to which they are applied. The ETAR method, for example, has been shown to yield an accuracy of better than 3% in lung dose calculations.^(11)^

The second method of accounting for tissue inhomogeneity employs model‐based radiation transport within the heterogeneous medium. This approach results in the calculation of absolute dose that may be converted to a relative distribution using normalization at a suitable point. The AAA falls broadly into this later category. It employs spatially invariant scatter kernels derived from Monte Carlo calculations and separately models primary photons, extrafocal photons, and contaminant electrons. The AAA does not, however, directly model secondary electron transport. Tissue heterogeneity is handled by radiologic scaling of primary photons and photon scatter kernel scaling in lateral directions according to local electron density. (For an excellent overview of the AAA, the reader is referred to a paper by Van Esch et al.^(12)^)

The degree of accuracy exhibited by dose distributions generated by the AAA has been examined by a number of authors.^(^ ^12^ ^–^ ^14^ ^)^ In particular, measurements by Van Esch et al.^(12)^ using an anthropomorphic phantom indicate that the AAA may overpredict the dose to regions distal to lung by as much as 6% for 6 MV and 4% for 15 MV. In pursuit of a full accounting of all factors affecting delivered dose, it is often desirable to model radiation transport not only within the heterogeneous tissues comprising the human body, but also through air gaps and patient‐related appliances such as head rests, immobilization devices, and retracted compensators. To that end, the present article examines a spectrum of materials, ranging from air, through intermediate‐density materials (of densities lesser and greater than water), to a high‐density bone‐equivalent analog. In this study, the specific ability of the AAA to accurately account for the presence of inhomogeneities composed of these materials in simple geometries is examined. Previous studies of the AAA and its prediction of dose distal to regions of heterogeneity have focused on a limited number of materials (lung and bone) with physical sizes near the maximal extent of clinical relevance. The present study examines a more complete sampling of clinically relevant densities and spatial dimensions. The source of the discrepancies observed with the AAA distal to heterogeneous regions has yet to be addressed in the literature. The goal of 2% accuracy set by AAPM TG‐65 serves as a useful benchmark against which to evaluate the inhomogeneity correction capabilities of this treatment planning algorithm.

## II. METHODS AND MATERIALS

**Figure 1:**
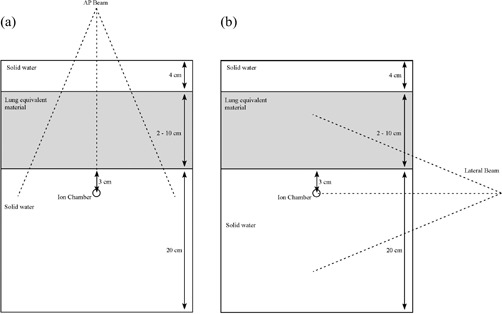
Experimental setup for (a) anterior–posterior (AP) beam arrangement, and (b) lateral beam arrangement.

**Figure 2:**
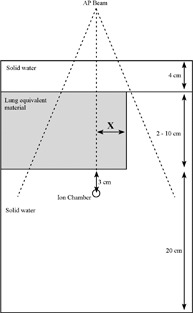
Experimental setup for an inhomogeneity layer of variable lateral extent [anterior–posterior (AP) beam arrangement].

The ability of the AAA to accurately model heterogeneity in simplified geometric circumstances was tested using the planar geometries depicted in Figs. 1 and 2. The experimental setup consisted of 3 layers of Solid Water, which constitute the homogeneous state. The topmost layer was 4 cm in thickness, the middle layer was of variable thickness, and the final layer was 20 cm in thickness. The heterogeneous condition was created by substituting, one at a time, materials of equivalent thickness, but of different composition, for the middle layer. The materials used for the heterogeneity layer were air, a lung‐equivalent material (Gammex RMI), foam Polyvinylchloride (fPVC), an adipose‐tissue analog (Scanpias: St. Bartholomew's Hospital, London, U.K.), polyvinyl chloride (PVC), and aluminum, spanning the range of densities that occur naturally within or immediately surrounding the human body. The thicknesses of these materials were chosen according to clinical relevance and constrained by local availability. Table 1 lists their pertinent properties. The measurement point in all cases was located at isocenter on the beam central axis at a vertical depth of 3 cm below the proximal surface of the heterogeneity layer. All measurements were taken using a Protea ion chamber (Protea Systems Corporation, Benicia, CA) in combination with a Capintec 192 electrometer (Capintec, Pittsburgh, PA). The cylindrical Protea ion chamber, with an active volume of 0.1 cm^3^ (1.2 cm in length, 0.2 cm inner diameter, and 0.325 cm outer diameter), was inserted into a form‐fitting channel milled into the bottom layer of Solid Water. An equal number of monitor units (MUs) was delivered in heterogeneous and homogeneous cases alike, and measured inhomogeneity correction factors $\left({ICF}_{meas}\right)$ were experimentally determined as the ratio of electrometer readings in those two states:

(1) $$ICF_{meas}=\frac{electrometer\;reading\;(heterogeneous\;conditions)}{electrometer\;reading\;(homogeneous\;conditions)}.$$

The error bars that appear in the graphs in this article are the result of standard propagation of errors stemming from the uncertainty associated with each individual measurement in the heterogeneous and the homogeneous cases alike. A minimum of 2 electrometer readings were obtained for each combination of beam energy, field size, material type, and material thickness. From these readings, the mean electrometer reading and its associated limit error were calculated. To address instances that yielded a limit error of zero value (identical electrometer readings), a larger number of readings (n=10) were obtained for small (5×5 cm), medium (10×10 cm), and large (20×20 cm) field sizes for each combination of beam energy, material type, and material thickness. The standard deviation for each set of 10 readings was determined, and the mean of those 3 values was calculated. The greater of the limit error and this mean deviation value (for each combination of beam energy, material type, and material thickness) was assigned as the error associated with each data point.

Three different arrangements were utilized for the experimental measurements.

The first was an anterior‐posterior (AP) setup in which the beam was directed vertically downward orthogonal to the plane of the various layers [see Fig. 1(a)]. Here, the heterogeneity layer extends in all directions beyond the lateral extent of the beam. The ICFs were measured as a function of field size and thickness of the inhomogeneity layer. In this arrangement, the ICF is influenced by both altered primary attenuation along the central ray path and change in scattered radiation to the measurement point introduced by the inhomogeneity layer.

In the second setup, the beam was rotated by 90 degrees, to a lateral orientation [Fig. 1(b)], and ICFs were measured as a function of field size for various thicknesses of lung‐equivalent material. Here, the primary beam path remains unaltered, and the ICF is influenced only by the change in scattered radiation to the measurement point.

**Table 1:** Pertinent properties of inhomogeneity layer materials

*Material*	*Physical density* $\left(g/cm^{3}\right)$	*Electron density (relative to water)*	CT# *(HU)*	*Thickness of material used (cm)*
Air	$1.2\times 10^{-3}$	$1.1\times 10^{-3}$	−1000	2, 4, 6, 8, 10
Lung‐equivalent material	0.27	0.265	−750	2, 4, 6, 8, 10
Foam PVC	0.61	0.56	−430	2, 4, 6, 8, 10
Adipose‐tissue equivalent	0.92	0.93	−100	2, 4, 6, 8, 10
PVC	1.40	1.29	545	1.3, 2.6, 3.9
Aluminum	2.70	2.34	2200	1.3, 2.6, 3.9

PVC = Polyvinylchloride.

Finally, in a third arrangement, the beam was again set in the AP direction, but the field size was fixed at 20×20 cm, and the thickness of the inhomogeneity layer, at 6 cm. In this configuration, the lateral extent of the inhomogeneity layer in the *x* direction is varied from x=−9.0 cm to *x* x=+9.0 cm in increments of 2 cm (Fig. 2). For values of x<0, the attenuation along the primary beam path, as seen by the measurement point, is unaltered. For values of x>0, the ICF is influenced by both altered primary and scattered radiation. This arrangement thus allows for the observation of a transition between a state in which scattered radiation alone influences the ICF and one in which alterations in both primary and scattered radiation play a role.

All measurements were performed for both 6‐MV and 15‐MV photon beams. The 6‐MV measurements were obtained using a Varian 600C (Varian Medical Systems) linear accelerator (LINAC) while a Varian 2300CD LINAC was used for the 15‐MV measurements. The 2300CD LINAC is capable of generating both 6‐MV and 15‐MV beams, but greater accessibility to the 600C LINAC was available, thus prompting the aforementioned measurement segregation. Field sizes up to a maximum of 25×25 cm were investigated for all low‐density $\left(\rho <1.0\;g/{cm}^{3}\right)$ inhomogeneity materials. Because of the size limitations imposed by materials availability, maximum field sizes were restricted to 15×15 cm for the high‐density $\left(\rho >1.0\;g/{cm}^{3}\right)$ heterogeneities.

These experimental setups were modeled within the Eclipse TPS running AAA version 7–5–18. The system was set to determine the number of MUs required to deliver an identical prescription dose to the measurement point in homogeneous and heterogeneous cases alike. The ICF as predicted by the AAA $\left({ICF}_{AAA}\right)$ is then given by

(2) $$ICF_{AAA}=\frac{required\;monitor\;units\;(homogeneous\;conditions)}{required\;monitor\;units\;(heterogeneous\;conditions)}.$$

The geometry used in Eclipse was created using phantom creation routines. Physically scanning a rectangular phantom produces images corrupted by small but discernable artifacts that result from the sharp corners. Within the phantom, these artifacts take the form of subtle variations in computed tomography (CT) number that would serve only to obfuscate the analysis. Creating the phantom within Eclipse itself eliminates these uncertainties. The luxury of a slight excess of materials allowed for the machining of cylindrical samples of each solid material. The CT number used for each type of material was determined by physically scanning the various samples. To ensure the greatest fidelity in CT number, each sample was centered within a cylindrical water phantom. The CT‐number‐to‐electron‐density lookup table used in Eclipse for all calculations was the one used clinically at the author's institute and was derived from the same scanner used to image the study samples. The CT numbers for the Solid Water and the lung‐equivalent material were specified by their respective manufacturers. The CT numbers derived by physical measurement agreed very well (within 1%) with manufacturer‐stated values.

## III. RESULTS AND DISCUSSION

**Figure 3:**
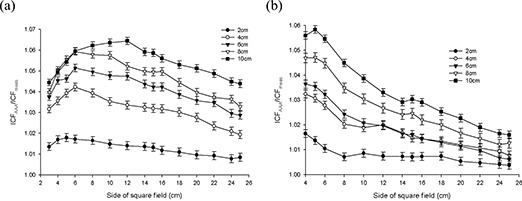
Anterior–posterior inhomogeneity correction factor (ICF) ratio [analytic anisotropic algorithm (AAA)–calculated/measured] as a function of field size and air inhomogeneity layer thickness at (a) 6 MV, and (b)15 MV.

**Figure 4:**
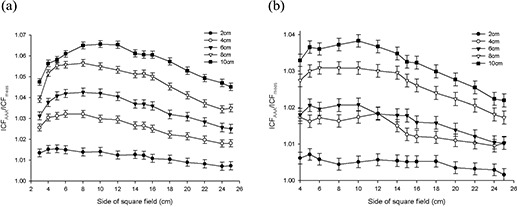
Anterior–posterior inhomogeneity correction factor (ICF) ratio [analytic anisotropic algorithm (AAA)–calculated/measured] as a function of field size and lung equivalent material inhomogeneity layer thickness at (a) 6 MV, and (b) 15 MV.

**Figure 5:**
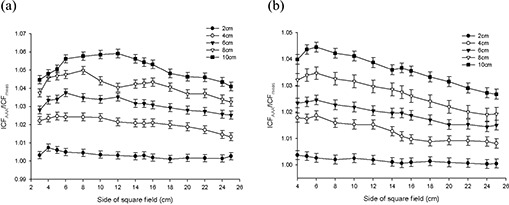
Anterior–posterior inhomogeneity correction factor (ICF) ratio [analytic anisotropic algorithm (AAA)–calculated/measured] as a function of field size and foam polyvinylchloride inhomogeneity layer thickness at (a) 6 MV, and (b) 15 MV.

**Figure 6:**
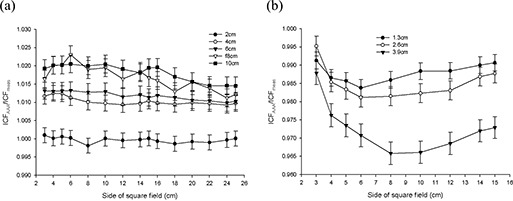
(a) Anterior–posterior inhomogeneity correction factor (ICF) ratio [analytic anisotropic algorithm (AAA)–calculated/ measured] as a function of field size and adipose‐tissue analog inhomogeneity layer thickness at 6 MV. (b) Anterior– posterior inhomogeneity correction factor (ICF) ratio [analytic anisotropic algorithm (AAA)–calculated/measured] as a function of field size and aluminum inhomogeneity layer thickness at 6 MV.

Figs. 3 – 6 present the results from the AP field arrangement in Fig. 1(a) as ICF ratios (AAA‐calculated to measured: ${ICF}_{AAA}/{ICF}_{meas}$) plotted as a function of field size and thickness of inhomogeneity material. (For the sake of brevity, only data sets that reveal discrepancies of greater than 2% between ${ICF}_{AAA}$ and ${ICF}_{meas}$ for this arrangement are presented graphically.) The following trends are observed:

- For low‐density $\left(\rho \leq 1.0\;g/{cm}^{3}\right)$ materials (air, lung‐equivalent, fPVC, and the adipose‐tissue analog) ${ICF}_{AAA}\geq {ICF}_{meas}$, with the reverse $\left({ICF}_{AAA}\leq {ICF}_{meas}\right)$ being true for the high‐density $\left(\rho \geq 1.0\;g/{cm}^{3}\right)$ heterogeneity materials (PVC and aluminum). These results correspond to overpredictions and underpredictions respectively of the dose distal to these non‐unit density regions.
- The discrepancy between ${ICF}_{AAA}$ and ${ICF}_{meas}$ increases as the thickness of the heterogeneity layer increases for each material. These ratios $\left({ICF}_{AAA}/{ICF}_{meas}\right)$ range from unity or near unity (within experimental error) for the thinnest heterogeneity layers to 1.065 for 10 cm of air and 0.966 for 3.9 cm of aluminum.
- At 6 MV for air, the lung‐equivalent material, fPVC, and aluminum, the field size at which the maximum deviation between ${ICF}_{AAA}$ and ${ICF}_{meas}$ occurs increases as the thickness of the material increases. No discernable trend could be identified in the results obtained with the adipose‐tissue analog and the PVC material. A similar trend could not be identified with the 15‐MV data.

Results at 6 MV with air, the lung‐equivalent material, and fPVC clearly demonstrate that the AAA‐calculated ICFs exceed their measured values by more than 2% for thicknesses of 6 cm and greater for all field sizes examined. A deviation of more than 2% from measured values is also seen for a thickness of 4 cm for field sizes of less than 24×24 cm for air, 22×22 cm for lung, and 12×12 cm for fPVC. The AAA‐calculated ICFs agree with their true measured values within 2% for all field sizes when 2 cm of the foregoing three materials formed the heterogeneity layer. The adipose‐tissue analog and PVC yielded better than 2% agreement (within experimental error) between calculated and measured ICFs for all thicknesses and all field sizes examined. Agreement within 2% between AAA‐calculated and measured ICFs also resulted for all field sizes examined for 1.3 cm and 2.6 cm of aluminum, but only for the smallest field size (3×3 cm) when 3.9 cm of aluminum was examined.

When 15‐MV photons were used, agreement within 2% was achieved between AAA‐calculated and measured ICFs for all field sizes and material thicknesses for aluminum, PVC, and adipose‐tissue analog. Agreement within 2% for all field sizes was also obtained for 2 cm of air; 2 cm and 4 cm of fPVC; and 2 cm, 4 cm, and 6 cm of lung‐equivalent material. For 10 cm of both fPVC and lung‐equivalent material, the disagreement between the AAA‐calculated and measured values was always greater than 2%. Mixed results were obtained with 6 cm and 8 cm of fPVC and 8 cm of lung‐equivalent material. For air, agreement to within 2% (within experimental error) was achieved for all thicknesses at the largest field size tested (25×25 cm). However, the level of agreement diminished as field sizes decreased, exceeding 2% for thicknesses of 4 cm, 6 cm, 8 cm, and 10 cm for field sizes less than 6×6 cm, 8×8 cm, 16×16 cm, and 22×22 cm respectively. Overall, the results obtained at 15 MV are significantly improved as compared with those obtained with 6 MV.

Despite the improvements seen at 15 MV, both data sets clearly demonstrate the inability of the AAA to model simple heterogeneous geometries to better than 2% accuracy over a significant range of clinically relevant dimensions and densities. However, what cannot be determined from these data alone is whether the observed failures stem from improper modeling of altered primary attenuation, the inability to accurately handle scattered radiation in the heterogeneous state, or a combination of those two effects.

**Figure 7:**
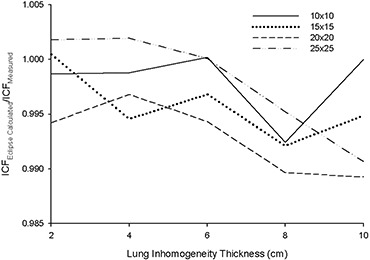
Lateral inhomogeneity correction factor (ICF) ratio [analytic anisotropic algorithm (AAA)–calculated/measured] as a function of field size and lung‐equivalent material inhomogeneity layer thickness at 6 MV.

Fig. 7 presents results from the lateral field arrangement in Fig. 1(b) for 6‐MV photons. (A similar trend is observed at 15MV.) Here, for both energies, most of the ICFs calculated by the AAA are less than or equal to the measured values. The AAA is seen to overestimate slightly the reduction in dose adjacent to a region of low density. In all cases, the differences between AAA‐calculated and measured ICF values are less than 1.1% and indicate that the effects of scatter are well modeled by the AAA—at least in this simple geometric arrangement. These results are opposite to the effects seen in the preceding AP beam arrangement, suggesting that deficiencies in handling primary attenuation are the root cause of the AAA‐related errors in the calculation of dose in regions distal to heterogeneous regions.

**Figure 8:**
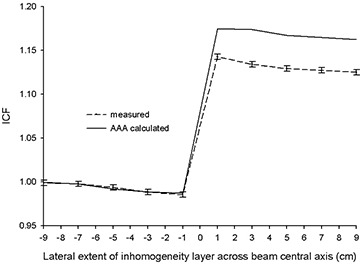
Inhomogeneity correction factors [ICFs; analytic anisotropic algorithm (AAA)–calculated and measured] as a function of lateral extent (*x* direction) of inhomogeneity layer (lung‐equivalent material, 6 cm) across beam central axis for 6 MV.

**Figure 9:**
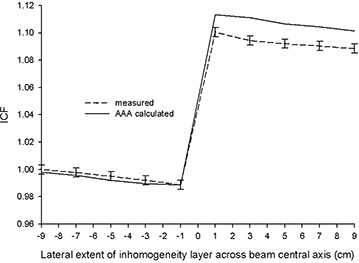
Inhomogeneity correction factors [ICFs; analytic anisotropic algorithm (AAA)–calculated and measured] as a function of lateral extent (*x* direction) of inhomogeneity layer (lung‐equivalent material, 6 cm) across beam central axis for 15 MV.

Figs. 8 and 9 show the results obtained with the AP beam arrangement in Fig. 2, where the field size and inhomogeneity layer thickness and material are constant, but the lateral extent of the lung‐equivalent inhomogeneity varied. A clear trend is seen at both energies. Agreement within experimental error is seen between AAA‐calculated and measured ICFs in all instances in which the inhomogeneity layer does not extend past the beam central axis. The AAA tracks, within experimental error, the changing scatter conditions, which cause a small but discernable reduction in measured ICFs as the lateral extent of the inhomogeneity layer increases before crossing the central beam axis. As would be expected, measured ICFs increase dramatically when the inhomogeneity crosses the beam central axis because of decreased primary attenuation. At both energies, the AAA‐calculated ICFs lie above their corresponding measured values for all x>0. Although exceeding their true measured values, the AAA‐calculated and measured ICFs both exhibit the same relative trend of a second moderate reduction in magnitude as the lateral extent of the inhomogeneity layer further increases.

The observations from the AP beam orientation of Fig. 2, in combination with the data from the lateral beam arrangement of Fig. 1(b), strongly indicate that the deficiencies revealed with the AP beam configuration of Fig. 1(a) are the result of a mishandling by the AAA of primary beam attenuation along heterogeneous beam paths. This result is rather surprising, because changes in primary attenuation are generally considered easier to correct than are alterations in scattered radiation. Unfortunately, the proprietary nature of commercial TPSs prevents an inspection of the actual code involved. That limitation effectively reduces these systems to “black box” status in terms of an exact determination of the root cause of the anomalies observed in the output.

The net results of these deviations are, however, immediately discernable. When primary beam paths encounter heterogeneities, the AAA will incorrectly predict dose distal to those deviations from unit density. The magnitude of the effect increases as tissue densities depart further and further from unity. Moreover, errors are greater with low‐density tissues than they are with comparable quantities of high‐density material. In particular, even relatively small path lengths of 4 cm through lung can result in inaccuracies of 3% at 6 MV. Ray paths through lung of up to, and sometimes exceeding, 10 cm are not uncommon in radiotherapy planning for pulmonary cancer, possibly resulting in errors approaching 7% at 6 MV and 4% at 15 MV. The same conclusions apply equally to air gaps. Moreover, when accounting for the presence of base plates used with headrests and other similar appliances (which often involve the inclusion of significant amounts of low‐ to medium‐density materials such as the foam PVC used in this investigation), AAA‐related errors of up to 6% can be expected. From the data gathered with aluminum at 6 MV, which may serve as a reasonable analog to bone, errors in AAA‐calculated ICFs exceeded 2% only for the largest thickness (3.9 cm) of that material investigated. No errors exceeding 1% were observed at 15 MV for all amounts of aluminum tested (including 3.9 cm in thickness).

The results presented here accord with the findings of Van Esch et al.,^(12)^ which revealed the AAA capable of substantial overestimations of the dose beyond low‐density heterogeneities. Furthermore, an examination by Fogliata et al.^(13)^ of the influence of inhomogeneities on depth dose and profile characteristics as modeled by four planning systems [Varian Eclipse (AAA and pencil‐beam convolution), Nucletron Helax‐TMS (Nucletron, Veenendaal, Netherlands), Philips Pinnacle (Philips Medical Systems, Andover, MA), and CMS XiO (CMS, St. Louis, MO)] reveals a general trend of overprediction by the AAA of dose within low‐density regions. Those results, taken together with the findings presented here, raise concerns regarding the ability of the AAA to adequately handle the presence of heterogeneities for radiotherapy treatment planning. Subjecting all new TPSs to a standard battery of rigorous tests before clinical release may prove extremely advantageous to those faced with the daunting tasks of comparing results produced by different algorithms and of planning for future acquisitions.

## IV. CONCLUSIONS

The ability of the AAA to correctly account for the presence of inhomogeneities in simple geometric circumstances was assessed by comparison with experiments at 6 MV and 15 MV photon energies. In general, the AAA was found to overpredict dose beyond low‐density heterogeneities and to underpredict dose beyond high‐density heterogeneities. In the case of low‐density heterogeneities, use of the AAA‐indicated number of MUs for actual treatment delivery would result in a dose deficiency with an accompanying reduction in expected tumor control. Where high densities are involved, concerns arise about the possibility of elevated normal‐tissue complications. In either case, the inability of the AAA to properly handle the heterogeneous state could well produce a negative therapeutic consequence. The magnitude of the discrepancies observed between AAA and experiment were found to be greater for 6 MV than for 15 MV. The largest discrepancies associated with each of these photon energies are associated with low‐density regions. If the AAPM TG‐65 benchmark of better than 2% accuracy in correcting for inhomogeneities is used, the AAA is seen to fail in numerous circumstances that can readily arise in the conduct of radiotherapy treatment planning in both its conventional and more leading‐edge expressions (conformal, intensity‐modulated, image‐guided, and adaptive). The source of these failures appears to arise from an inability to correctly account for altered attenuation along primary ray paths, rather than from deficiencies in modeling scattered radiation.

## Supporting information

Supplementary Material

Supplementary Material

Supplementary Material

Supplementary Material

Supplementary Material

Supplementary Material

Supplementary Material
