# Supplementary material for: Inhomogeneity correction and the analytic anisotropic algorithm
Source: J Appl Clin Med Phys. 2008 May 1;9(2):112–22. doi: 10.1120/jacmp.v9i2.2786 (PMC5721710; doi:10.1120/jacmp.v9i2.2786)
Supplement: Supplementary file 1 — Supplementary Material [file ACM2-9-112-s001.doc]

INHOMOGENEITY CORRECTION AND

THE ANALYTIC ISOTROPIC ALGORITHM (AAA)

**Don Robinson^1,2^**

*^1^Department of Medical Physics, Cross Cancer Institution, 11560 University Ave, Edmonton, Canada, T6G*

*^2^Department of Oncology, University of Alberta, 11560 University Ave, Edmonton, Canada, T6G 1Z2*

Corresponding Author:

**Don Robinson, Ph.D.**

*Department of Medical Physics*

*Cross Cancer Institute*

*11560 University Ave, Edmonton, Alberta, Canada T6G 1Z2*

[*don.robinson@cancerboard.ab.ca*](mailto:don.robinson@cancerboard.ab.ca)

“Inhomogeneity Correction and the AAA”
