# Supplementary material for: Inhomogeneity correction and the analytic anisotropic algorithm
Source: J Appl Clin Med Phys. 2008 May 1;9(2):112–22. doi: 10.1120/jacmp.v9i2.2786 (PMC5721710; doi:10.1120/jacmp.v9i2.2786)
Supplement: Supplementary file 2 — Supplementary Material [file ACM2-9-112-s002.doc]

**ABSTRACT**

The ability of the analytic isotropic algorithm (AAA), a superposition-convolution algorithm implemented in the Eclipse (Varian Medical Systems, Palo Alto, California) treatment planning system (TPS), to accurately account for the presence of inhomogeneities in simple geometries is examined. The goal of 2% accuracy, as set out by the American Association of Physicists in Medicine (AAPM) Task Group 65, serves as a useful benchmark against which to evaluate the inhomogeneity correction capabilities of this treatment planning algorithm. A planar geometry phantom, consisting of upper and lower layers of solid water separated by a heterogeneity region of variable thickness, is modeled within the Eclipse TPS. Results obtained with the AAA are compared to experimental measurement. Seven different materials, used one at a time, ranging in density from air to aluminum, constitute the inhomogeneity layer. In general, the AAA is seen to over predict dose beyond low density regions while under prediction characterizes the output distal to volumes of high density. In many cases the deviation between AAA and experimental results exceeds the Task Group 65 target of 2%. The source of these deviations appears to arise from an inability of the AAA to correctly account for altered attenuation along primary ray paths.

Key Words: inhomogeneity correction factor, Analytic isotropic algorithm, AAA
