# Supplementary figures and images for: Inhomogeneity correction and the analytic anisotropic algorithm
Source: J Appl Clin Med Phys. 2008 May 1;9(2):112–22. doi: 10.1120/jacmp.v9i2.2786 (PMC5721710; doi:10.1120/jacmp.v9i2.2786)

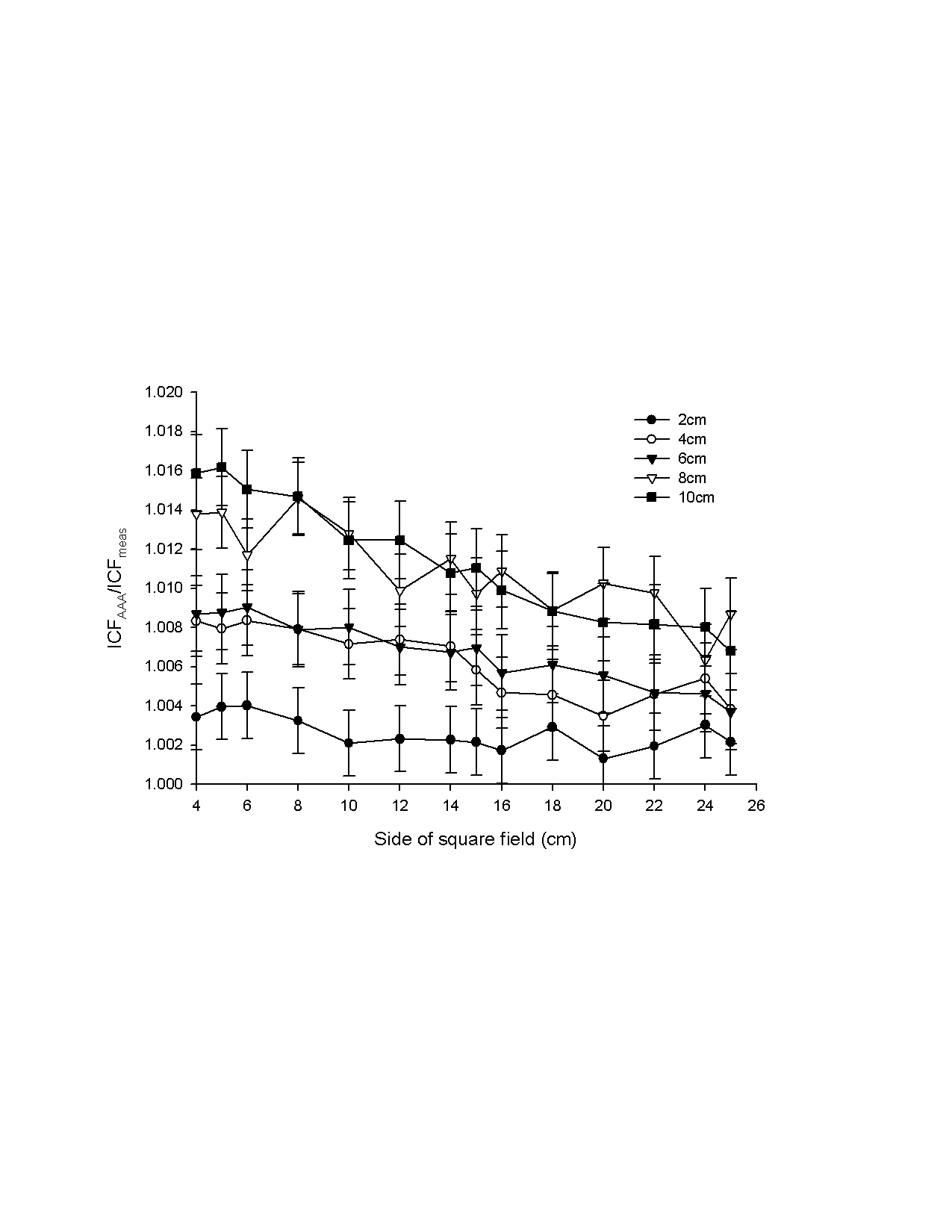

Supplement: Supplementary file 3 — Supplementary Material [file ACM2-9-112-s003.jpg]

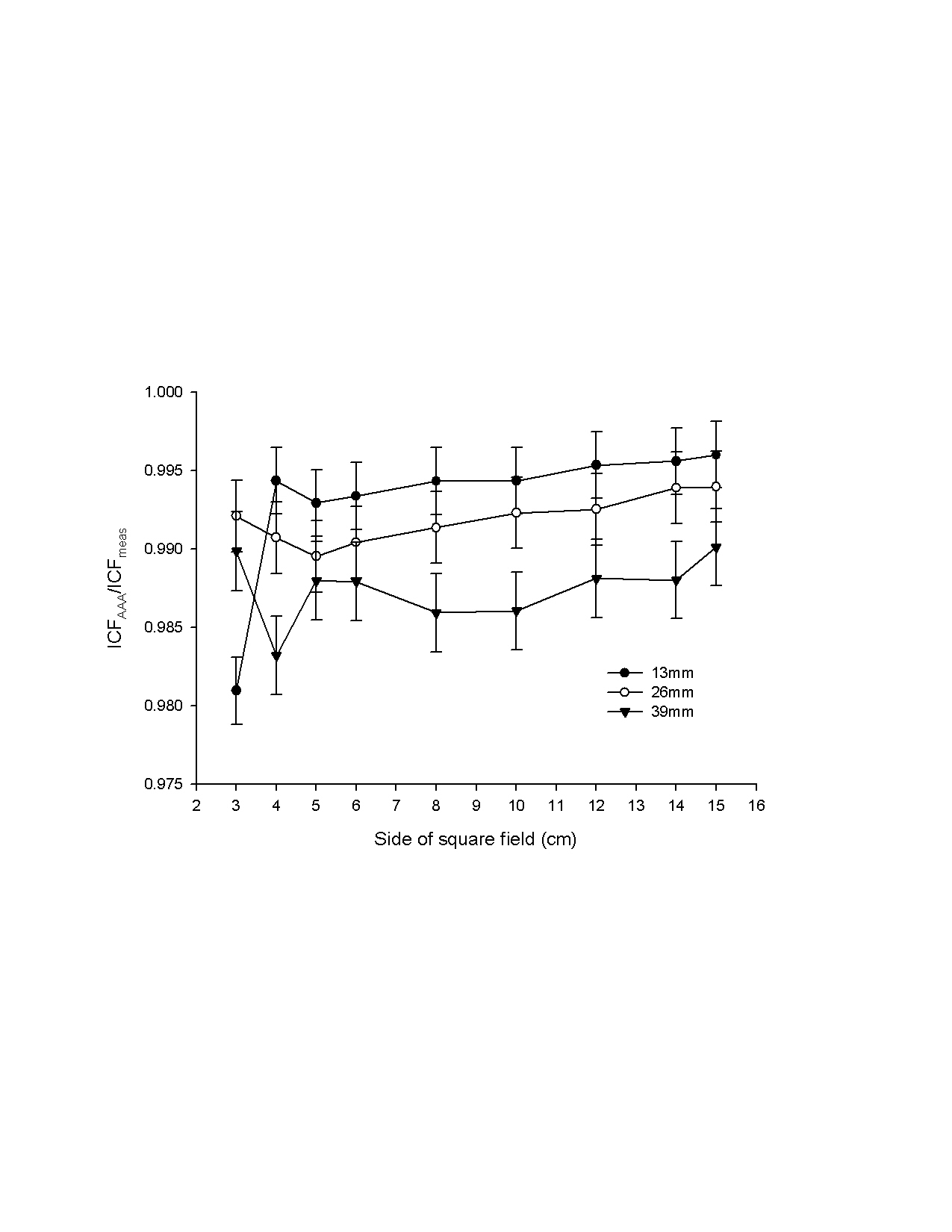

Supplement: Supplementary file 4 — Supplementary Material [file ACM2-9-112-s004.jpg]

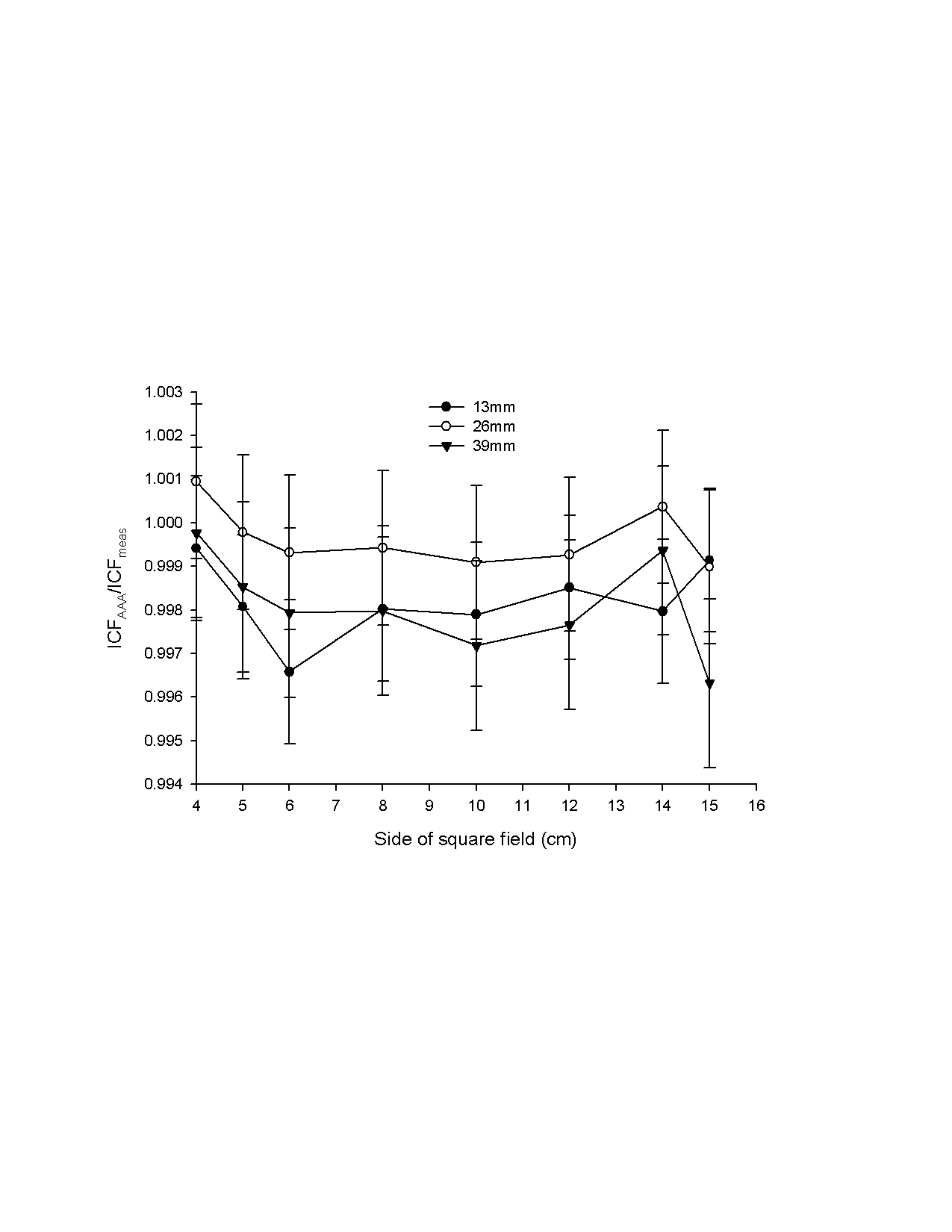

Supplement: Supplementary file 5 — Supplementary Material [file ACM2-9-112-s005.jpg]

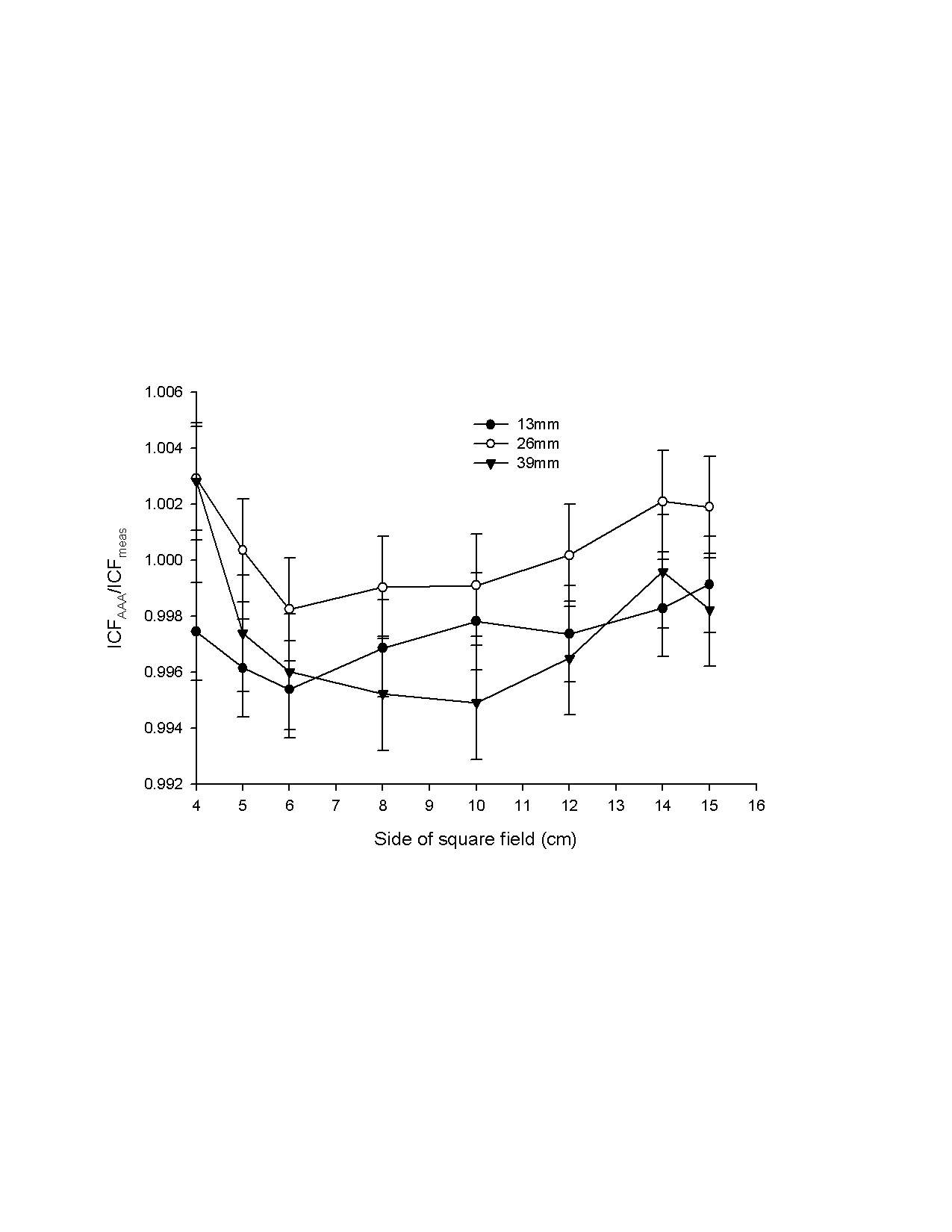

Supplement: Supplementary file 6 — Supplementary Material [file ACM2-9-112-s006.jpg]

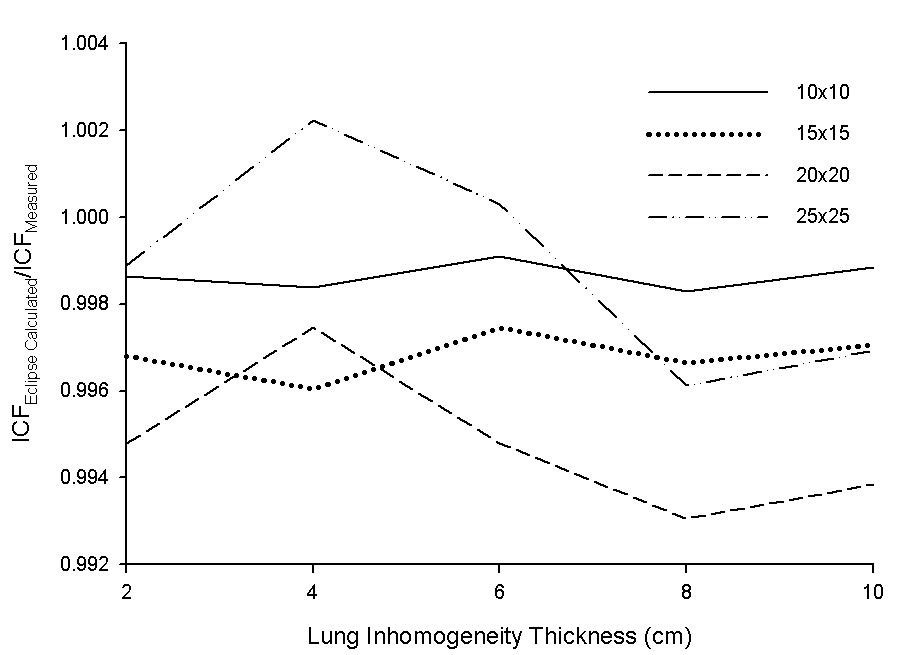

Supplement: Supplementary file 7 — Supplementary Material [file ACM2-9-112-s007.jpg]
